# Exercise training upregulates SIRT1 to attenuate inflammation and metabolic dysfunction in kidney and liver of diabetic *db/db* mice

**DOI:** 10.1186/s12986-019-0349-4

**Published:** 2019-04-02

**Authors:** Hung-Wen Liu, Hao-Han Kao, Chi-Hang Wu

**Affiliations:** Department of Physical Education, National Taiwan Normal University, 162, Section 1, Heping E. Rd, Taipei City, Taiwan

**Keywords:** Moderate exercise, SIRT1, NF-κB, Mitochondrial function, Diabetic db/db mice

## Abstract

**Background:**

Chronic inflammation and metabolic dysregulation may eventually cause tissue damage in obesity-related diseases such as type 2 diabetes. The effects of SIRT1 on integration of metabolism and inflammation may provide a therapeutic target for treatment of obesity-related diseases. We examined the underlying mechanism of moderate intensity aerobic exercise on kidney and liver in obese diabetic *db/db* mice, mainly focusing on inflammation and metabolic dysfunction.

**Methods:**

Functional and morphological alterations and metabolic and inflammatory signaling were examined in type 2 diabetic *db/db* mice with or without exercise training (5.2 m/min, 1 h/day, and 5 days/week for a total of 8 weeks).

**Results:**

Exercise training prevented weight gain in *db/db* + Ex mice, but it did not reduce glucose and insulin levels. Exercise lowered serum creatinine, urea, and triglyceride levels and hepatic AST and ALT activity in *db/db* + Ex mice. Reduced kidney size and morphological alterations including decreased glomerular cross-sectional area and hepatic macrovesicles were observed in *db/db* + Ex mice compared with untrained db/db mice. Mechanistically, preventing loss of SIRT1 through exercise was linked to reduced acetylation of NF-κB in kidney and liver of *db/db* + Ex mice. Exercise increased citrate synthase and mitochondrial complex I activity, subunits of mitochondrial complexes (I, II, and V) and PGC1α at protein level in kidney of *db/db* + Ex mice compared with non-exercise db/db mice. Changes in enzyme activity and subunits of mitochondrial complexes were not observed in liver among three groups.

**Conclusion:**

Exercise-induced upregulation of SIRT1 attenuates inflammation and metabolic dysfunction, thereby alleviating the progression of diabetic nephropathy and hepatic steatosis in type 2 diabetes mellitus.

## Background

Chronic inflammation and metabolic dysregulation may eventually cause tissue damage in metabolic diseases, particularly obesity, type 2 diabetes mellitus (T2DM), and cardiovascular disease [1]. Under pathophysiological conditions, mitochondrial dysfunction results in overproduction of mitochondrial reactive oxygen species (ROS) and further stimulates nuclear factor-kappa B (NF-κB) activity, thus leading to cellular damage and tissue dysfunction [2, 3]. Sirtuin 1 (SIRT1), a NAD+ dependent deacetylase, functions as an energy sensor and integrates cellular metabolism and inflammation via regulating downstream signaling pathways [4]. Therefore, downregulation of SIRT1 may be one of the underlying mechanisms of disease progression [5]. Loss of SIRT1 is associated with upregulation of peroxisome proliferator-activated receptor gamma coactivator 1-α (PGC-1α) and NF-κB acetylation, thereby impairing mitochondrial biogenesis and triggering inflammation in T2DM [6]. Previous studies have demonstrated that activation of SIRT1 improves diabetes-related chronic kidney disease [7] and nonalcoholic fatty liver disease [8]. The effect of SIRT1 on integration of metabolism and inflammation may provide a therapeutic target for treatment of kidney disease and fatty liver disease.

Diabetic animal models reveal protective effects of exercise in several tissues including kidney [9–12], liver [13], skeletal muscle [13, 14], and blood vessels [15], however protective effect of exercise dose not completely rely on its glucose-lowering effects. SIRT1 regulates metabolism and inflammation in various tissues [4] which can be the key regulator of exercise-mediated protection against diabetes at tissue level. In addition to skeletal muscle [14], the protective effects of long-term exercise training in the regulation of inflammation and metabolic dysfunction via SIRT1 signaling pathway have been less investigated in peripheral tissues such as kidney and liver. Obese diabetic db/db mice exhibit albuminuria, podocyte loss, and mesangial matrix expansion in kidney [16] along with hepatic lipid accumulation [17]; the mouse model is often used in T2DM research. Treadmill exercise often used in animal models of obesity and diabetes, but intensive exercise may lead to unfavorable outcome [18]. Elevated cortisol levels is associated with failure of glycemic control in diabetic *db/db* mice performed high intensity exercise (15 m/min for 30 min) [19]. Low intensity aerobic exercise (5.2 m/min for 60 min) might be more appropriate exercise prescription than high intensity for diabetic *db/db* mice to reduce hormonal-metabolic stress [10, 14].

We hypothesized that moderate intensity aerobic exercise training delays the progression of diabetic nephropathy and hepatic steatosis by restoring SIRT1-mediated metabolic and inflammatory signaling in *db/db* mice, a model of T2DM.

## Methods

### Materials

Primary antibodies: Inhibitor of kappa B alpha (IκBα) (#4814), NF-κB p65 (#4764), phospho-NF-κB p65 (Ser536) (#3033), and β-actin (#4967) were purchased from Cell signaling (Danvers, MA). Acetyl-NFκB (Lys310) (ab19870), NDUFB8 (ab110242), CII-30 kDa (ab14714), CIII-Core protein2 (ab14745), CIV subunit1 (ab14705), CVα subunit (ab14748), PGC1α (ab54481), and SIRT1 (ab12193) were purchased from Abcam (Cambridge, MA). Goat anti-rabbit (#7074) and horse anti-mouse (#7076) HRP conjugated secondary antibodies were purchased from Cell signaling (Danvers, MA).

### Experimental animals

Four-week-old male diabetic C57BLKS/J (*db/db*) mice (*n* = 16) and their age-matched controls (*m/m*, *n* = 8) were purchased from the National Laboratory Animal Center (Taipei, Taiwan). Animal experiments were approved by the National Taiwan Normal University Institutional Animal Care and Use Committee (Approval Number: 105030). Two mice per cage were housed in an air-conditioned animal facility at 20 ± 2 °C, 50 ± 5% humidity, and 12 h light/dark cycle with free access to water and normal chow diet (LabDiet 5058, St. Louis, MO, USA). After 1 week acclimatization, *db/db* mice were divided randomly into two groups: *db/db* (*n* = 8) remained sedentary throughout the study and *db/db* + Ex group (*n* = 8) received 8-week moderate exercise training. Animals were anesthetized by intraperitoneal injection of urethane (1500 mg/1 kg BW) followed by decapitation between 10 to 12 pm. Trunk blood was collected from overnight fasted mice in nonheparinized tubes. Serum was separated by centrifugation at 3000 rpm for 15 min and stored at − 20 °C. Kidneys were removed, briefly rinsed with PBS, removed excess fluid and fat, and weighed on a digital balance. Left kidney and left lobe of liver were fixed with 4% paraformaldehyde. Right kidney and the remaining liver tissue were stored at − 80 °C for further analysis.

### Exercise training

Moderate exercise training protocol used in the present study has been shown to increase citrate synthase activity, a marker of the skeletal muscle oxidative adaptation to aerobic exercise training, in *db/db* mice [10]. Eight weeks moderate-intensity exercise (5.2 m/min, 1 h/day, and 5 days/week for a total of 8 weeks) was started from 5-wk-old [14]. During the first week, mice ran on a motorized treadmill (30 min with 0° slope) and exercise duration was gradually increased from 30 min to the target of 1 h (0° slope). Mice were exercised at 9-11 am. *db/db* and *m/m* mice remained sedentary were placed on the treadmill belt for the same duration.

### Biological markers

Diluted blood sample were measured by ACCU-CHEK blood glucose meter (Roche, Basel, Switzerland). Serum insulin and tumor necrosis factor α (TNFα) were measured by Milliplex® map kit (Millipore, Billerica, MA, USA). For measurement of creatinine level, serum samples (25 μl, 5 times diluted) were mixed with assay buffer, creatinase, creatininase, enzyme mix, and creatinine probe, incubated 60 min at 37 °C, and then read at 570 nm. For measurement of urea level, serum samples (25 μl, 50 times diluted) were mixed with assay buffer, OxiRed probe, developer, enzyme mix, and converter enzyme, incubated 60 min at 37 °C, and then read at 570 nm. For measurement of triglyceride (TG) levels, serum samples (5 μl) were mixed with TG assay buffer, TG probe, and enzyme mix, incubated 60 min at room temperature, and then read at 570 nm [20]. Levels of creatinine, urea, and TG were calculated following manufacturer’s instructions (BioVision, Milpitas, CA, catalog#K375, K625, and K622).

### Renal and hepatic histology

Histological examination was performed in the National Laboratory Animal Center (Taipei, Taiwan). Embedded liver blocks from *m/m*, *db/db*, and *db/db* + Ex groups (*n* = 4/group) were cut into 5 μm sections and stained with hematoxylin-eosin. Embedded kidney blocks from *m/m*, *db/db*, and *db/db* + Ex groups (*n* = 4/group) were cut into 5 μm sections and stained with Periodic acid–Schiff (PAS). Images were observed under a microscope and captured with a digital camera (Canon Inc., Tokyo, Japan). Average glomerular area of kidney was determined using ImageJ with careful manual annotations and 20–25 glomeruli per animal were counted.

### Hepatic AST and ALT activity

For measurement of aspartate aminotransferase (AST) and alanine aminotransferase (ALT) activity, liver samples (~ 10 mg) were homogenized in ice-cold assay buffer. Tissue homogenates were mixed with assay buffer, enzyme mix, developer, and substrate, incubated 60 min at 37 °C, and then measured in a microplate spectrophotometer (DYNEX, Chantilly, VA, USA). Hepatic enzyme activity was calculated following manufacturer’s instructions (BioVision, Milpitas, CA, USA, catalog#K752 and 753) according to the manufacturer’s instructions [20].

### Western blot analyses

Liver and kidney were cut into small pieces and homogenized in ice-cold RIPA buffer containing 1 mM phenylmethylsulfonyl fluoride and protease inhibitor cocktail (Millipore, Billerica, MA). Total protein in the homogenate was measured by the Bradford dye-binding method (Bio-Rad, Hercules, CA). Homogenates of liver and kidney were separated by SDS-PAGE, transferred to nitrocellulose membrane, and incubated with appropriate antibodies. Protein bands were visualized using chemiluminescence kit (Millipore, Billerica, MA) and quantified by using the LAS-4000 mini biomolecular imager (GE HealthCare Life Sciences, Pittsburgh, PA, USA).

### Mitochondrial enzyme activity

Crude mitochondrial fraction was extracted from gastrocnemius muscle, kidney, and liver using mammalian mitochondrial isolation kit (BioVision, Milpitas, CA, USA, catalog#K288) followed by manufacturer’s instructions. Protein concentration was measured by the Bradford dye-binding method (Bio-Rad, Hercules, CA, USA). For measurement of citrate synthase activity in muscle, kidney, and liver, mitochondrial extract (5 μg) was mixed with assay buffer, developer, and substrate mix and then immediately read at 412 nm for 40 min at 5 min interval. For measurement of mitochondrial complex I activity in kidney and liver, mitochondrial extract (5 μg) was mixed with assay buffer, decylubiquinone, and dye and then immediately read at 600 nm for 5 min at 30s interval. For measurement of mitochondrial complex IV activity, mitochondrial extract (5 μg) was mixed with reduced cytochrome c and then immediately read at 550 nm for 30 min at 30 s interval [14]. Citrate synthase activity, mitochondrial complex I (NADH:ubiquinone oxidoreductase) activity, and IV (cytochrome oxidase) activity were calculated following manufacturer’s instructions (BioVision, Milpitas, CA, catalog#K318, K968, and K287).

### Statistical analysis

Data are expressed as means ± SEM. The statistical significance of the differences among *m/m*, *db/db*, and *db/db* + Ex groups was determined by one-way ANOVA and following post hoc assessment by Student-Newman-Keuls Method correction for multiple comparisons (SigmaPlot 12.0, San Jose, CA, USA). Different lowercase letters indicate significant differences among groups.

## Results

### Effects of exercise training on body weight, glucose, insulin, and TNFα

**Fig. 1:**
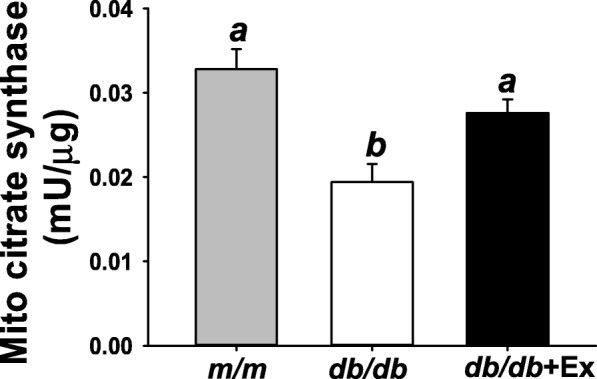
Effect of moderate exercise training on citrate synthase in gastrocnemius muscle. Mitochondrial citrate synthase activity in gastrocnemius muscle (*n* = 8/group). Values presented are mean ± SEM. Significance (*P* < 0.05) among groups is denoted by different letters

Citrate synthase activity in skeletal muscle is used as a marker of physiological adaptation to aerobic exercise training. Moderate exercise training increased mitochondrial citrate synthase activity in gastrocnemius muscle of *db/db* + Ex group compared with non-exercise group (Fig. 1). Body weight in *db/db* and *db/db* + Ex groups were higher than m/m mice (Table 1). A mild reduction of body weight (− 7.0%) was observed in *db/db* + Ex mice compared with non-exercise *db/db* mice (Table 1). Blood glucose and serum insulin levels have been shown to be increased 6- to 7- and 3- to 4-fold, respectively, in *db/db* mice compared with *m/m* mice (Table 1). Blood glucose and serum insulin levels were not affected by aerobic exercise training (Table 1). Raising low levels of serum TNFα was observed in *db/db* + Ex compared with non-exercise group (Table 1).

**Table 1:** General parameters

	*m/m*	*db/db*	*db/db* + Ex
Body weight (g)	24.5 ± 0.6^a^	44.4 ± 1.1^b^	41.3 ± 1.3^c^
Glucose (mg/dl)	85 ± 4^a^	551 ± 37^b^	605 ± 29^b^
Insulin (pg/ml)	1353 ± 90^a^	5077 ± 786^b^	5842 ± 755^b^
TNFα (pg/ml)	6.4 ± 0.4^a^	3.7 ± 0.3^b^	4.8 ± 0.3^c^

Values are mean ± SEM (*n* = 8). Significance (*P* < 0.05) among groups is denoted by different letters

### Effects of exercise training on kidney and liver

**Fig. 2:**
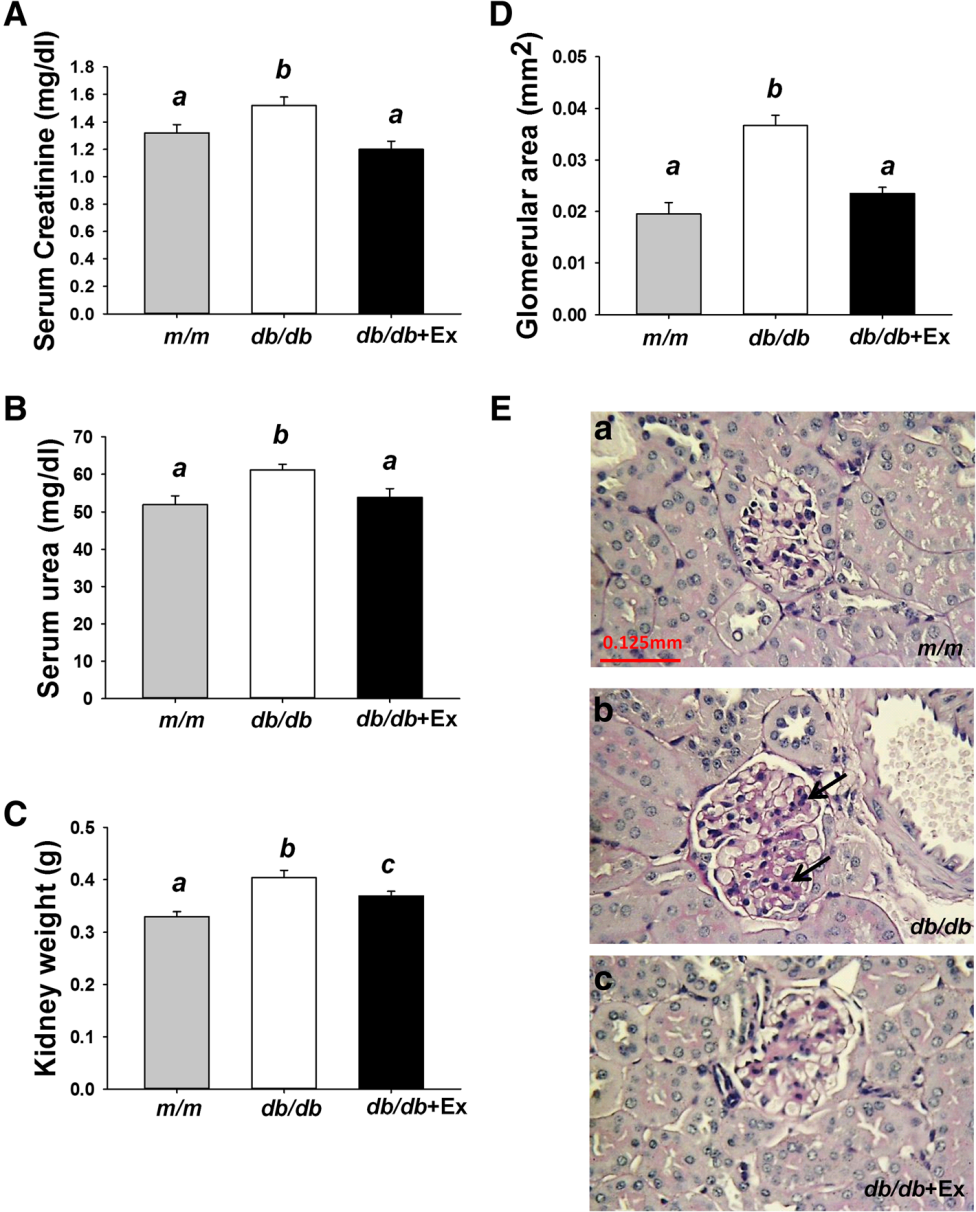
Effect of moderate exercise training on kidney function and morphology. Serum levels of creatinine (**a**) and urea (**b**) and kidney weight (**c**, *n* = 8/group). Mean glomerular area of kidney (**d**, *n* = 4/group). PAS-stained kidney sections (**e**) from *m/m* (*a*), *db/db* (*b*), and *db/db* + Ex mice (*c*) (400X). Arrows point to the modest mesangial expansion seen in *db/db* mice. Values presented are mean ± SEM. Significance (*P* < 0.05) among groups is denoted by different letters

Serum creatinine and urea levels are used as an index of renal function. Raised serum creatinine and urea levels were observed in diabetic *db/db* mice compared with *m/m* mice (Fig. 2a, b). Kidney weight and glomerular area were significantly increased in diabetic *db/db* mice (0.33 vs 0.4 g; 0.0195 vs 0.0367mm^2^) compared with *m/m* mice as shown in Fig. 2 c-e. Exercise decreased serum creatinine and urea levels, renal size and glomerular area in *db/db* + Ex group (Fig. 2a-e).

**Fig. 3:**
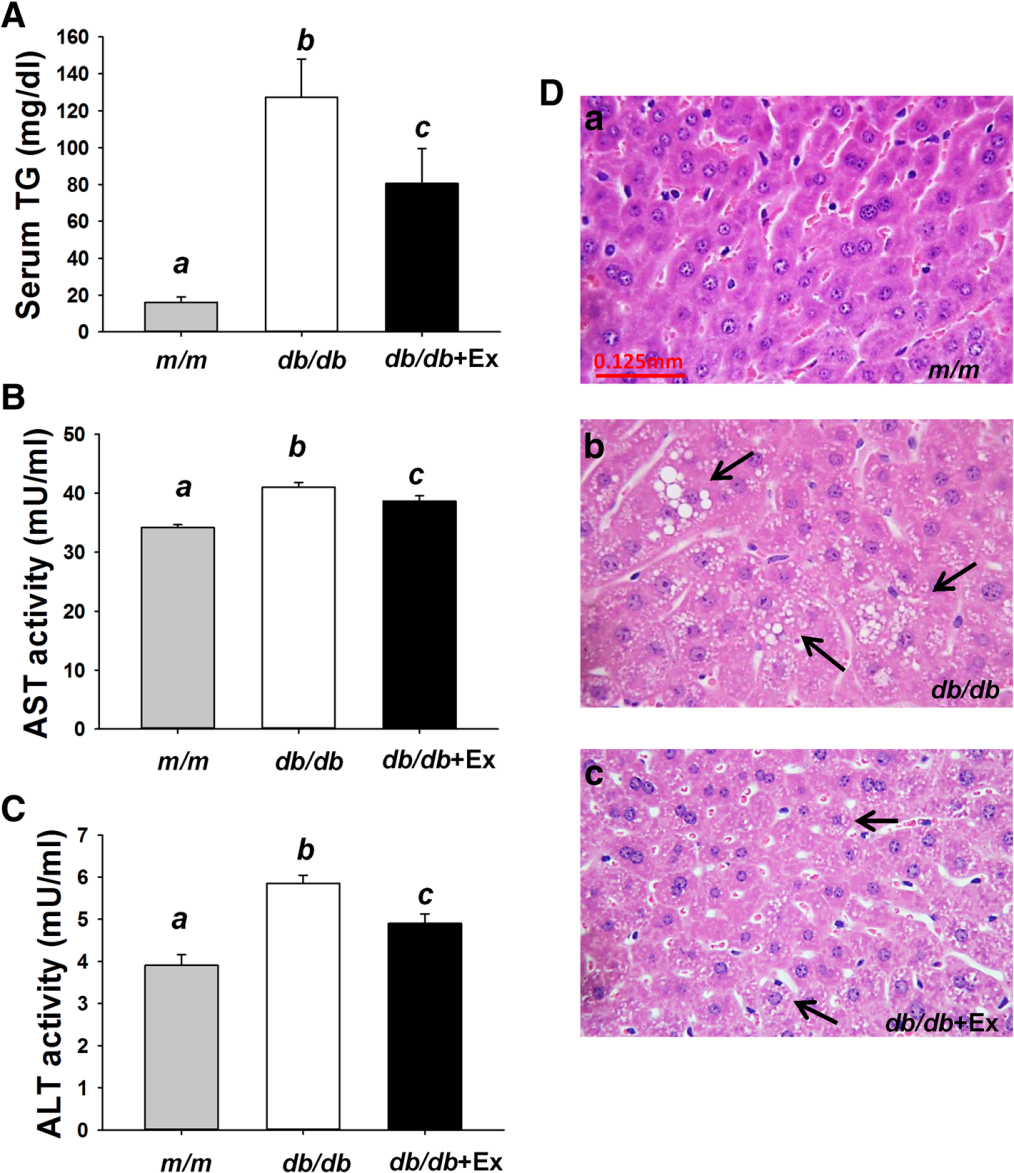
Effect of moderate exercise training on serum TG, liver function, and morphology. Serum levels of TG (**a**), hepatic AST (**b**) and ALT (**c**) activity (*n* = 8/group). Hematoxylin and eosin-stained liver sections (**d**) from *m/m* (*a*), *db/db* (*b*), *db/db* + Ex mice (*c*) (400X). Arrows indicate the accumulation of TG as macro or micro vesicles

Elevated liver enzyme activity such as ASL and ALT may indicate hepatic inflammation or damage. Increased serum TG levels, elevated hepatic AST and ALT activity, and displayed macro and micro vesicles (Fig. 3a-d) in liver were observed in *db/db* mice compared with m/m mice. Exercise attenuated serum TG levels and hepatic AST and ALT activity in *db/db* + Ex group (Fig. 3a-c). Exercise specifically decreased macro vesicles but had minimal effects on micro vesicles in liver of *db/db* + Ex group (Fig. 3 d).

### Effects of exercise on metabolic inflammation; SITR1 and NFκB signaling

**Fig. 4:**
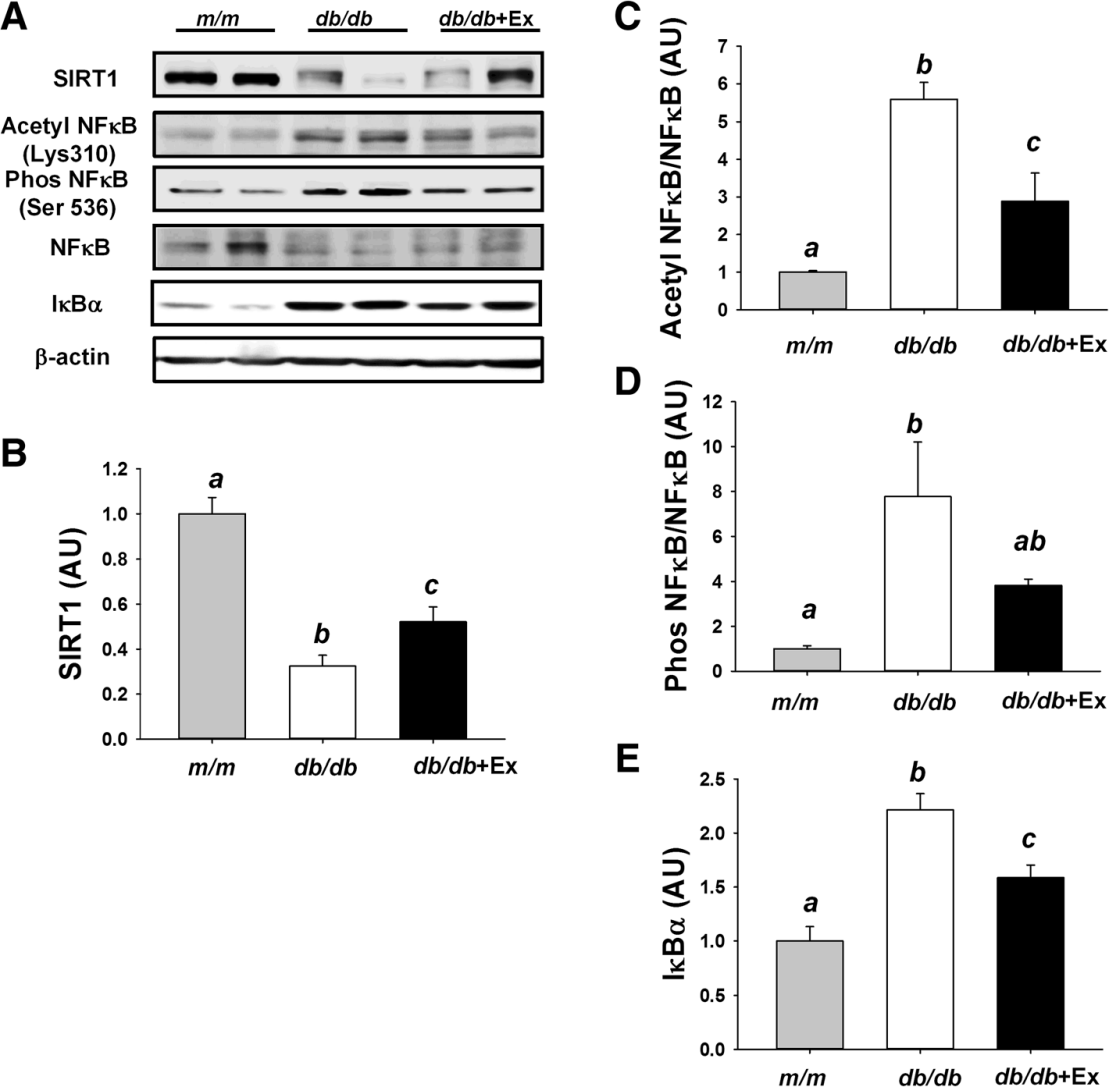
Effect of moderate exercise training on SIRT1/NF-κB signaling pathway in kidney. Representative blots of SIRT1, acetyl-NF-κB p65 (Lys310), phospho-NF-κB p65 (Ser536), NF-κB p65, and IκBα are shown (**a**). A quantitative bar graph of SIRT1 (**b**), acetyl NF-κB/NF-κB (**c**), phos NF-κB/NF-κB (**d**), and IκBα (**e**). Protein levels in kidney are presented mean ± SEM (*n* = 6/group). Significance (*P* < 0.05) among groups is denoted by different letters. Data are presented as fold change in protein levels normalized to *m/m* mice

SIRT1, a master regulator of energy metabolism, is associated with exercise training-induced mitochondrial biogenesis. SIRT1 also exhibits an anti-inflammatory effect through deacetylation of NF-κB, thus preventing nuclear translocation of NF-κB and pro-inflammatory gene expression. Decreased SIRT1 expression, increased acetylation and phosphorylation of NF-κB and were observed in kidney of diabetic *db/db* mice compared with non-diabetic *m/m* mice (Fig. 4a-d). Downregulation of SIRT1 and activation of NF-κB via acetylation and phosphorylation were partially normalized by moderate exercise training in kidney of *db/db* + Ex mice (Fig. 4a-d). Compared with non-diabetic *m/m* mice, *db/db* mice showed increased expression of IκBα, while exercise decreased IκBα expression in *db/db* + Ex group (Fig. 4e).

**Fig. 5:**
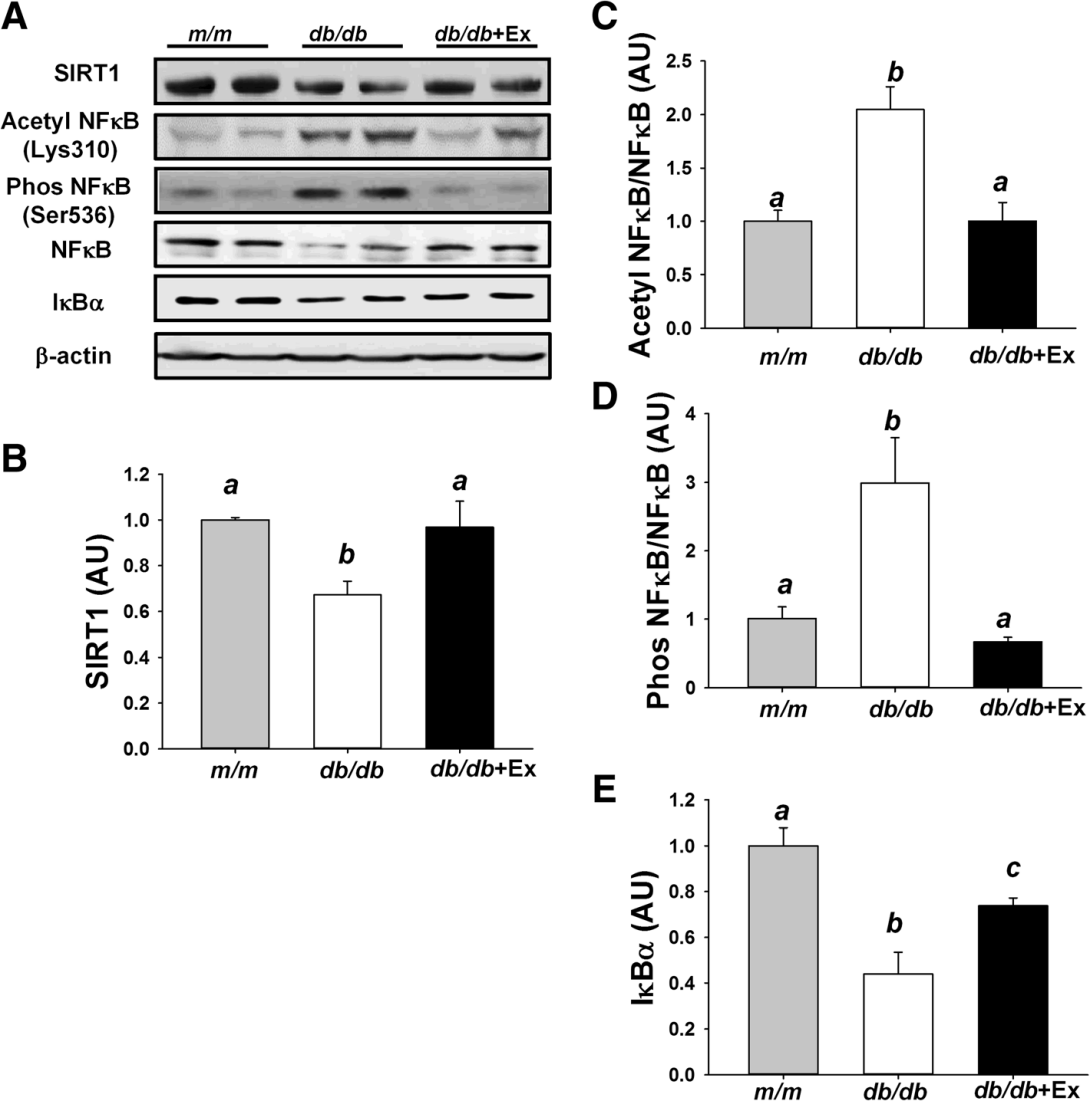
Effect of moderate exercise training on SIRT1/NF-κB signaling pathway in liver. Representative blots of SIRT1, acetyl-NF-κB p65 (Lys310), phospho-NF-κB p65 (Ser536), NF-κB p65, and IκBα are shown (**a**). A quantitative bar graph of SIRT1 (**b**), acetyl NF-κB/NF-κB (**c**), phos NF-κB/NF-κB (**d**), and IκBα (**e**). Protein levels in liver are presented mean ± SEM (*n* = 6/group). Significance (*P* < 0.05) among groups is denoted by different letters. Data are presented as fold change in protein levels normalized to *m/m* mice

Reduced SIRT1 and IκBα expression was associated with increased acetylation and phosphorylation of NF-κB in liver of *db/db* mice compared with *m/m* mice (Fig. 5a-e). Exercise training inhibited acetylation and phosphorylation of NF-κB via upregulating SIRT1 and IκBα expression in liver of db/db + Ex mice (Fig. 5a-e).

### Effects of exercise training on mitochondrial function in kidney and liver

**Fig. 6:**
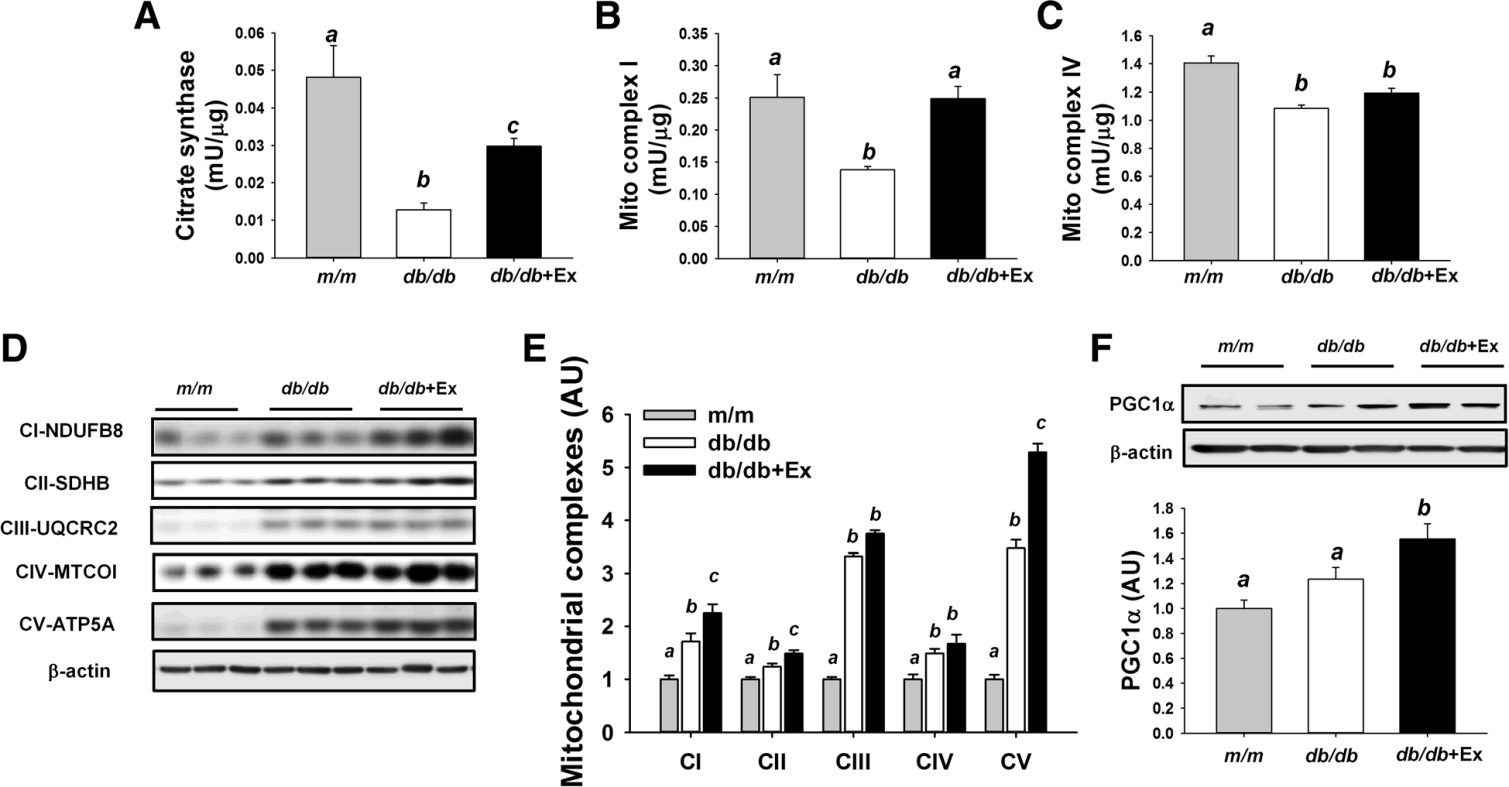
Effect of moderate exercise training on mitochondrial function in kidney. Mitochondrial citrate synthase activity (**a**) and mitochondrial complex I and IV activity in kidney (**b**-**c**, *n* = 8/group). Representative blots of mitochondrial complex I, II, III, IV, V, and PGC1α (**d**, **f**) are shown. The protein levels in kidney are presented mean ± SEM (**e-f**, *n* = 6/group). Significance (*P* < 0.05) among groups is denoted by different letters. Data are presented as fold change in protein levels normalized to *m/m* mice

Mitochondrial dysfunction contributes to the early development of diabetic kidney disease and fatty liver disease. Decreased enzyme activity including citrate synthase, NADH: coenzyme Q oxidoreductase (mitochondrial complex I), and cytochrome c oxidase (mitochondrial complex IV) and markedly increased subunits of mitochondrial complexes (I-V) were observed in kidney of *db/db* mice compared with *m/m* mice as shown in Fig. 6 a-e. Slightly increased PGC1α (+ 24%) was observed in kidney of diabetic *db/db* mice compared with non-diabetic m/m mice (*p* = 0.118, Fig. 6 f). Exercise training significantly increased citrate synthase and mitochondrial complex I activity, subunits of mitochondrial complexes (I, II, and V), and PGC1α at protein level in kidney of *db/db* + Ex mice compared with non-exercise *db/db* mice (Fig. 6a, b, d-f).

**Fig. 7:**
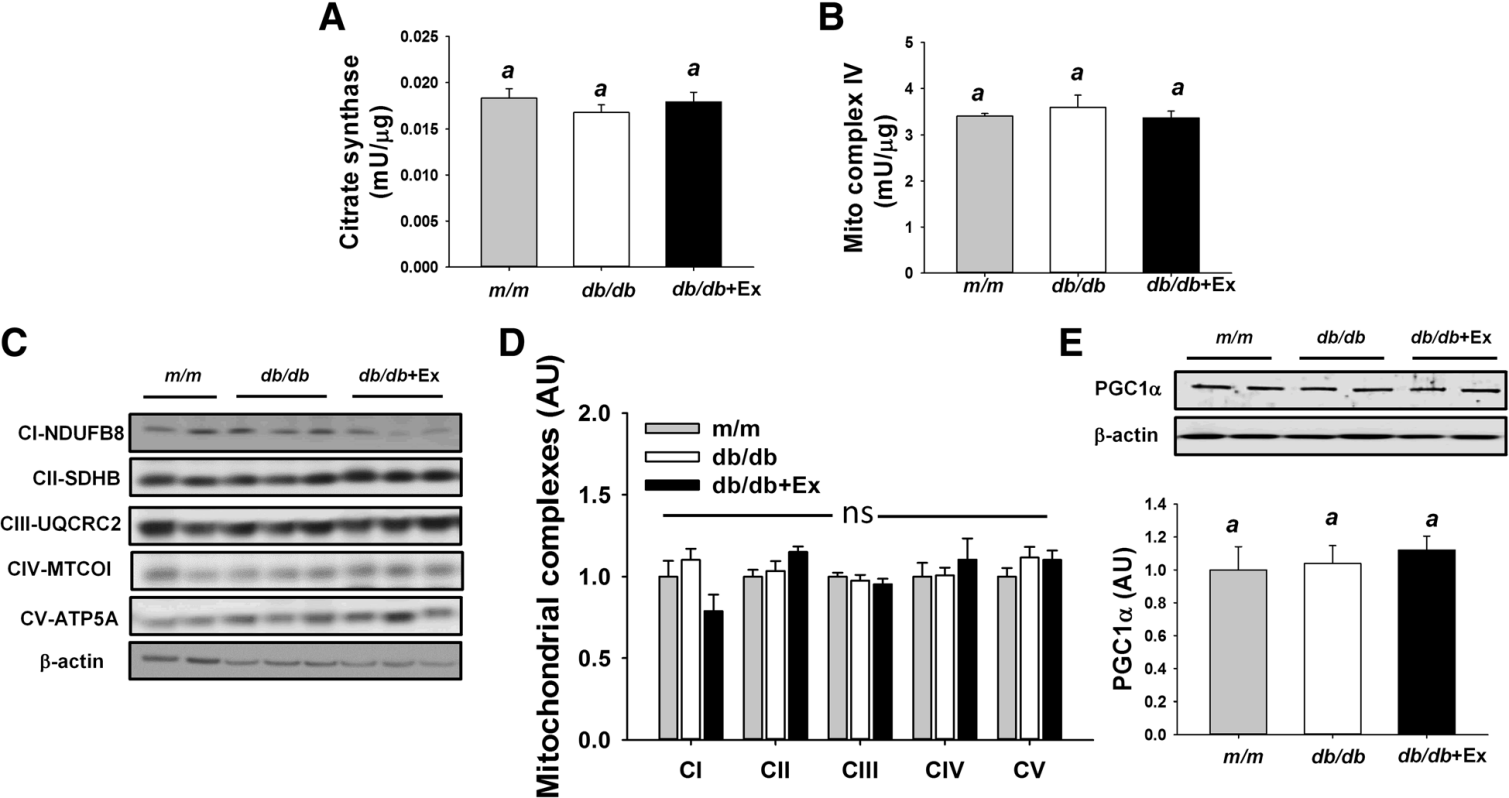
Effect of moderate exercise training on mitochondrial function in liver. Mitochondrial citrate synthase activity (**a**) and mitochondrial complex IV activity (**b**) in liver (*n* = 8/group). Representative blots of mitochondrial complex I, II, III, IV, V, and PGC1α (**c**, **e**) are shown. The protein levels in kidney are presented mean ± SEM (**d**-**e**, *n* = 6/group). Significance (*P* < 0.05) among groups is denoted by different letters. Data are presented as fold change in protein levels normalized to *m/m* mice

No differences in citrate synthase activity, mitochondrial complex IV activity, and subunits of mitochondrial complexes and PGC1α were observed in liver among three groups (Fig. 7a-e).

## Discussion

Hyperglycemia-induced oxidative stress is mainly responsible for the pathology of diabetic complications [21]. Aerobic exercise training has been considered as an effective treatment for the management of glycemic control in patients with T2DM. Nevertheless, recent evidence from several diabetic animal studies indicates that exercise exerts its protective effects on renal function [9, 10, 12], liver [13], muscle loss [14], and endothelial function [15] dependently or independently of glycemic control. The present study provides a novel molecular mechanism by which moderate exercise alleviates the progression of renal dysfunction and hepatic steatosis through SIRT1-mediated regulation of metabolism and inflammation in diabetic *db/db* mice.

Increasing evidence suggests that NF-κB activation is involved in the pathogenesis of diabetes-associated complications [22]. SIRT1, a NAD^+^ − dependent deacetylase, exhibits anti-inflammatory effects through NF-κB deacetylation [4]. Our results and other studies [23, 24] indicate that activation of NF-κB is associated with decreased SIRT1 expression in diabetic animal models. Exercise-induced upregulation of SIRT1 and inhibition of NF-κB acetylation were observed in the present study. Targeting NF-κB activation via restoration of SIRT1 expression for the modulation of acetylation status has been confirmed in diabetic rodent models by pyridoxamine treatment [23] or dietary restriction [6]. The results of this study support previous findings that exercise regulates tissue specific changes of SIRT1 and other sirtuins expression and activity in many tissues including skeletal muscle, brain, adipose tissue, and heart, thereby preventing metabolic diseases or aging-related disorders [25, 26].

In the canonical signaling pathway, IκBα phosphorylation and subsequently degradation can be triggered by intra- and extracellular stimuli such as ROS and/or TNFα, which leads to activation of NF-κB [22]. In the present study, the effect of exercise on IκBα/NF-κB signaling pathway in liver can be explained by the classic mechanism. On the other hand, renal overexpression of IκBα in *db/db* mice was not directly correlated with inhibition of NF-κB activity. Our results are in agreement with a previous study indicating that overexpression of IκBα is not associated with inhibition of NF-κB–DNA binding activity [27]. In addition, in vitro study demonstrates that phosphorylation of NF-κB at the Ser536 residue is not completely dependent on IκBα [28]. Last, but most important, attenuated overexpression of IκBα by exercise is linked to inhibition of NF-κB activity.

IkBα degradation is through ubiquitin-proteasome system [29]. Hyperglycemia impairs proteasome function in the diabetic kidney [30], whereas proteasome activity is enhanced in skeletal muscle of diabetic *db/db* mice [31]. Proteasome function is likely to alter differently in different tissues under certain pathological conditions. Taken together, the difference of IkBα expression between liver and kidney in *db/db* mice may depends on their rates of degradation.

Mitochondrial dysfunction including reduced mitochondrial biogenesis and depressed mitochondrial respiratory enzyme activity in skeletal muscle is the underlying molecular mechanism involved in the development of T2DM (19). Disrupted renal mitochondrial homeostasis may induce microvascular damage, promote inflammation and fibrosis, and consequently contribute to progression of diabetic nephropathy [32]. In the present study, exercise training restored renal function via activation of citrate synthase and NADH:ubiquinone oxidoreductase (complex I). Moreover, our data indicate that mitochondrial complex enzyme activity is correlative increased with mitochondrial complex expression in *db/db* + Ex mice. Exercise increases mitochondrial complex expression via the induction of PGC1α, a key regulator of mitochondrial biogenesis. In agreement with our finding, pharmacological activation of mitochondrial biogenesis by semisynthetic bile acid [24] or resveratrol, a chemical SIRT1 activator [33] has been shown to improve renal function in diabetic animal models. Therefore, targeting the pathway that regulates mitochondrial function is likely to prevent the progression of diabetic nephropathy. Furthermore, restored mitochondrial function by exercise training could prevent overproduction of mitochondrial ROS and subsequently suppress activation of NF-κB in kidney of diabetic *db/db* mice.

Increased mitochondrial biogenesis has been reported in kidney of *db/db* mice [34], which is in contrast to previous studies using different animal models [11, 33]. Increased renal gluconeogenesis, renal glucose uptake, and renal glucose uptake have been observed in T2DM [35], suggesting that kidney requires more mitochondria to produce energy to enable it to handle abnormal glucose metabolism. Therefore, increased mitochondrial biogenesis may be a physiological adaptation in response to high energy demand in kidney. In this context, increased mitochondrial complex expression may serve a protective role in the kidneys during early diabetic nephropathy.

In the present study, hepatic enzyme activity and mitochondrial complex expression were not affected at early age (13-wk-old), indicating that the progress of mitochondrial dysfunction is developed in a tissue-specific manner. Simultaneous comparison of mitochondrial markers in liver, glycolytic and oxidative muscle [36] and comparison of liver, muscle, and epididymal adipose tissue [37], studies have shown that changes in mitochondrial complexes at protein level are not uniformly altered in diabetic *db/db* mice compared with non-diabetic controls.

Impaired mitochondrial fatty acid oxidation is involved in the development and pathogenesis of steatosis [38]. Here, hepatic mitochondrial function in *db/db* mice remains intact at 13-wk-old. In line with our observation, normal hepatic mitochondrial respiratory capacity and citrate synthase activity as well as hepatic lipid accumulation have been observed concurrently in obese patients with and without type 2 DM compared with lean controls [39]. In the present case, mitochondrial dysfunction may not be the main factor involved in the development hepatic steatosis at this age. Exercise training attenuates hepatic lipid accumulation, at least in part, via lowering circulating triglyceride in diabetic *db/db* mice.

## Conclusion

The present study demonstrated the beneficial effects of moderate intensity aerobic exercise on kidney and liver function in diabetic *db/db* mice. Decreased SIRT1 expression is associated with increased NF-κB (p65) acetylation, whereas exercise represses NF-κB activity via the restoration of SIRT1 expression in kidney and liver. Furthermore, exercise induces mitochondrial complex expression via induction of PGC1α to improve enzyme activity in kidney. In conclusion, moderate intensity aerobic exercise is a promising intervention for counteracting metabolic dysregulation and inflammatory processes in type 2 DM.

## Abbreviations

ALT: Alanine aminotransferase
AST: Aspartate aminotransferase
IκBα: Inhibitor of kappa Bα
NF-κB: Nuclear factor-kappa B
PGC1α: Peroxisome proliferator-activated receptor gamma coactivator 1-α
ROS: Reactive oxygen species
SIRT1: Sirtuin 1
T2 DM: Type 2 diabetes mellitus
TG: Triglyceride

## References

[1] Hotamisligil GS (2006). Inflammation and metabolic disorders. Nature.

[2] Volpe CMO, Villar-Delfino PH, Dos Anjos PMF, Nogueira-Machado JA (2018). Cellular death, reactive oxygen species (ROS) and diabetic complications. Cell Death Dis.

[3] Hurrle S, Hsu WH (2017). The etiology of oxidative stress in insulin resistance. Biom J.

[4] Kauppinen A, Suuronen T, Ojala J, Kaarniranta K, Salminen A (2013). Antagonistic crosstalk between NF-kappaB and SIRT1 in the regulation of inflammation and metabolic disorders. Cell Signal.

[5] Kitada M, Koya D (2013). SIRT1 in type 2 diabetes: mechanisms and therapeutic potential. Diabetes Metab J.

[6] Kitada M, Takeda A, Nagai T, Ito H, Kanasaki K, Koya D (2011). Dietary restriction ameliorates diabetic nephropathy through anti-inflammatory effects and regulation of the autophagy via restoration of Sirt1 in diabetic Wistar fatty (fa/fa) rats: a model of type 2 diabetes. Exp Diabetes Res.

[7] Kong L, Wu H, Zhou W, Luo M, Tan Y, Miao L, Cai L (2015). Sirtuin 1: a target for kidney diseases. Mol Med.

[8] Colak Y, Yesil A, Mutlu HH, Caklili OT, Ulasoglu C, Senates E, Takir M, Kostek O, Yilmaz Y, Yilmaz Enc F (2014). A potential treatment of non-alcoholic fatty liver disease with SIRT1 activators. J Gastrointestin Liver Dis.

[9] Boor P, Celec P, Behuliak M, Grancic P, Kebis A, Kukan M, Pronayova N, Liptaj T, Ostendorf T, Sebekova K (2009). Regular moderate exercise reduces advanced glycation and ameliorates early diabetic nephropathy in obese Zucker rats. Metabolism.

[10] Ghosh S, Khazaei M, Moien-Afshari F, Ang LS, Granville DJ, Verchere CB, Dunn SR, McCue P, Mizisin A, Sharma K, Laher I (2009). Moderate exercise attenuates caspase-3 activity, oxidative stress, and inhibits progression of diabetic renal disease in db/db mice. Am J Physiol Renal Physiol.

[11] Gu Q, Zhao L, Ma YP, Liu JD (2015). Contribution of mitochondrial function to exercise-induced attenuation of renal dysfunction in spontaneously hypertensive rats. Mol Cell Biochem.

[12] Muhammad AB, Lokhandwala MF, Banday AA (2011). Exercise reduces oxidative stress but does not alleviate hyperinsulinemia or renal dopamine D1 receptor dysfunction in obese rats. Am J Physiol Renal Physiol.

[13] McGee-Lawrence ME, Wenger KH, Misra S, Davis CL, Pollock NK, Elsalanty M, Ding K, Isales CM, Hamrick MW, Wosiski-Kuhn M (2017). Whole-body vibration mimics the metabolic effects of exercise in male leptin receptor-deficient mice. Endocrinology.

[14] Liu HW, Chang SJ (2018). Moderate exercise suppresses NF-kappaB signaling and activates the SIRT1-AMPK-PGC1alpha Axis to attenuate muscle loss in diabetic db/db mice. Front Physiol.

[15] Moien-Afshari F, Ghosh S, Elmi S, Rahman MM, Sallam N, Khazaei M, Kieffer TJ, Brownsey RW, Laher I (2008). Exercise restores endothelial function independently of weight loss or hyperglycaemic status in db/db mice. Diabetologia.

[16] Alpers CE, Hudkins KL (2011). Mouse models of diabetic nephropathy. Curr Opin Nephrol Hypertens.

[17] Anstee QM, Goldin RD (2006). Mouse models in non-alcoholic fatty liver disease and steatohepatitis research. Int J Exp Pathol.

[18] Ghosh S, Golbidi S, Werner I, Verchere BC, Laher I (2010). Selecting exercise regimens and strains to modify obesity and diabetes in rodents: an overview. Clin Sci (Lond).

[19] Sennott J, Morrissey J, Standley PR, Broderick TL (2008). Treadmill exercise training fails to reverse defects in glucose, insulin and muscle GLUT4 content in the db/db mouse model of diabetes. Pathophysiology.

[20] Liu HW, Chan YC, Wang MF, Wei CC, Chang SJ (2015). Dietary (−)-Epigallocatechin-3-gallate supplementation counteracts aging-associated skeletal muscle insulin resistance and fatty liver in senescence-accelerated mouse. J Agric Food Chem.

[21] King GL, Loeken MR (2004). Hyperglycemia-induced oxidative stress in diabetic complications. Histochem Cell Biol.

[22] Patel S, Santani D (2009). Role of NF-kappa B in the pathogenesis of diabetes and its associated complications. Pharmacol Rep.

[23] Liu R, Zhong Y, Li X, Chen H, Jim B, Zhou MM, Chuang PY, He JC (2014). Role of transcription factor acetylation in diabetic kidney disease. Diabetes.

[24] Wang XX, Wang D, Luo Y, Myakala K, Dobrinskikh E, Rosenberg AZ, Levi J, Kopp JB, Field A, Hill A (2018). FXR/TGR5 dual agonist prevents progression of nephropathy in diabetes and obesity. J Am Soc Nephrol.

[25] Suwa M, Sakuma K (2013). The potential role of sirtuins regarding the effects of exercise on aging- related diseases. Curr Aging Sci.

[26] Pucci B, Villanova L, Sansone L, Pellegrini L, Tafani M, Carpi A, Fini M, Russo MA (2013). Sirtuins: the molecular basis of beneficial effects of physical activity. Intern Emerg Med.

[27] Emmerich F, Meiser M, Hummel M, Demel G, Foss HD, Jundt F, Mathas S, Krappmann D, Scheidereit C, Stein H, Dorken B (1999). Overexpression of I kappa B alpha without inhibition of NF-kappaB activity and mutations in the I kappa B alpha gene in reed-Sternberg cells. Blood.

[28] Sasaki CY, Barberi TJ, Ghosh P, Longo DL (2005). Phosphorylation of RelA/p65 on serine 536 defines an I {kappa}B {alpha}-independent NF-{kappa} B pathway. J Biol Chem.

[29] Chen Z, Hagler J, Palombella VJ, Melandri F, Scherer D, Ballard D, Maniatis T (1995). Signal-induced site-specific phosphorylation targets I kappa B alpha to the ubiquitin-proteasome pathway. Genes Dev.

[30] Queisser MA, Yao D, Geisler S, Hammes HP, Lochnit G, Schleicher ED, Brownlee M, Preissner KT (2010). Hyperglycemia impairs proteasome function by methylglyoxal. Diabetes.

[31] Wang X, Hu Z, Hu J, Du J, Mitch WE (2006). Insulin resistance accelerates muscle protein degradation: activation of the ubiquitin-proteasome pathway by defects in muscle cell signaling. Endocrinology.

[32] Bhargava P, Schnellmann RG (2017). Mitochondrial energetics in the kidney. Nat Rev Nephrol.

[33] Hui Y, Lu M, Han Y, Zhou H, Liu W, Li L, Jin R (2017). Resveratrol improves mitochondrial function in the remnant kidney from 5/6 nephrectomized rats. Acta Histochem.

[34] Kitada M, Kume S, Imaizumi N, Koya D (2011). Resveratrol improves oxidative stress and protects against diabetic nephropathy through normalization of Mn-SOD dysfunction in AMPK/SIRT1-independent pathway. Diabetes.

[35] Gerich JE (2010). Role of the kidney in normal glucose homeostasis and in the hyperglycaemia of diabetes mellitus: therapeutic implications. Diabet Med.

[36] Holmstrom MH, Iglesias-Gutierrez E, Zierath JR, Garcia-Roves PM (2012). Tissue-specific control of mitochondrial respiration in obesity-related insulin resistance and diabetes. Am J Physiol Endocrinol Metab.

[37] Choo HJ, Kim JH, Kwon OB, Lee CS, Mun JY, Han SS, Yoon YS, Yoon G, Choi KM, Ko YG (2006). Mitochondria are impaired in the adipocytes of type 2 diabetic mice. Diabetologia.

[38] Nassir F, Ibdah JA (2014). Role of mitochondria in nonalcoholic fatty liver disease. Int J Mol Sci.

[39] Lund MT, Kristensen M, Hansen M, Tveskov L, Floyd AK, Stockel M, Vainer B, Poulsen SS, Helge JW, Prats C, Dela F (2016). Hepatic mitochondrial oxidative phosphorylation is normal in obese patients with and without type 2 diabetes. J Physiol.

